# Effectiveness of Family-Centered Empowerment Model on Psychological Improvement of Patients With Myocardial Infarction: A Bayesian Multivariate Approach

**DOI:** 10.3389/fpubh.2022.878259

**Published:** 2022-07-11

**Authors:** Mehdi Raei, Mohammad Ghasemi, Kiavash Hushmandi, Nasrin Shirmohammadi-Khoram, Seyedeh Omolbanin Seyedrezaei, Hosein Rostami, Amir Vahedian-Azimi

**Affiliations:** ^1^Health Research Center, Life Style Institute, Baqiyatallah University of Medical Sciences, Tehran, Iran; ^2^Department of Epidemiology and Biostatistics, Faculty of Health, Baqiyatallah University of Medical Sciences, Tehran, Iran; ^3^Department of Food Hygiene and Quality Control, Division of Epidemiology & Zoonoses, Faculty of Veterinary Medicine, University of Tehran, Tehran, Iran; ^4^Department of Biostatistics, School of Public Health, Hamadan University of Medical Sciences, Hamadan, Iran; ^5^Department of Emergency Medicine, School of Medicine, Shahroud University of Medical Sciences, Shahroud, Iran; ^6^Trauma Research Center, Nursing Faculty, Baqiyatallah University of Medical Sciences, Tehran, Iran

**Keywords:** longitudinal, psychological, empowerment, myocardial infarction, bayesian

## Abstract

**Objective:**

There is a limited understanding of the impact of the family-centered empowerment model (FCEM) on the psychological symptoms in post-myocardial infarction (MI). This study aimed to evaluate the effectiveness of the FCEM on the psychological improvement of patients with MI.

**Methods:**

The present study was a randomized controlled trial (RCT) where patients experienced a standard home cardiac rehabilitation (CR) or CR utilizing the FCEM approach. The empowerment of patients was estimated during nine assessments, such as pre- and post-intervention. Factors, such as quality of life (QoL), state and trait anxiety, and perceived stress, were evaluated. A Bayesian multivariate mixed-effects model was used to simultaneously investigate the effect of the intervention group on study outcomes across the time.

**Results:**

Among all the participants in this study, 24 (34.3%) were women with a total mean ± standard deviation (SD) of 61.40 ± 12.83 and 24.87 ± 3.80 for age and body mass index (BMI). The participants who were in the FCEM group had a significantly higher mean level of perceived stress (β = 28.80), state anxiety (β = 16.20), trait anxiety (β = 3.65), physical (β = 38.54), and mental QoL (β = 42.14). Moreover, the individuals in the FCEM group had a significantly higher mean level of general health (β = 31.64) in the physical dimension of QoL, vitality (β = 15.04), mental role limitation (β = 21.84), and mental health (β = 18.16) in the mental dimension of QoL.

**Conclusions:**

The FCEM can be a valuable treatment mechanism for patients with post-MI to improve their stress, anxiety, and QoL.

## Introduction

Coronary heart disease (CHD) is a chronic situation, which presents a serious threat to human health, and has been occurring more frequently (1). Every 25 s, a new patient with CHD is diagnosed in the USA, and approximately 34% of these patients die every year. The mortality rate equals death per min (2, 3). Myocardial infarction (MI) not only has social, economic, health, and medical impacts but also it has psychological consequences that may challenge and threaten patients' mental health. Stress, anxiety, and poor quality of life (QoL) have been identified as common psychological consequences after MI (4).

Patients who have both cardiovascular disease and psychological problems attracted considerable attention. Psychological disorders, such as anxiety and stress, may lead to direct pathophysiological changes, increasing the risk of developing CHD (5). The study results showed that about 19–66% of patients with acute previous myocardial infarction (AMI) suffer from anxiety, which enhance the chances of mortality in them (6). In addition, patients with post-MI suffer from different physical and mental complications, such as weakness, energy loss, sleep disorders, fatigue, chest pain, and anxiety more, prevalently, which can restrict their daily activities (7). These complications have also adverse consequences on the progression of the disease (6–8). Moreover, according to previous studies, stress and anxiety in these patients affect all aspects of QoL in these patients (9, 10).

Cardiac rehabilitation (CR) is an outpatient chronic disease management model for secondary cardiovascular disease (CVD) prevention. It is a class I indication for patients with CHD (11). To develop physical capacity, CR outcomes involve improving sympathetic balance and improving mental competence (11, 12). According to a Cochrane review, psychological interventions may reduce psychological problems in cardiac patients (13). Although several approaches and educational programs have been applied in this context, they have not focused on the patient and their family or friends (14). Family-centered care interventions, in which a family member participates in each step of research, improve the capabilities of the family members in specific fields related to health and wellbeing, and thus they can overcome the existing obstacles (11). The family-centered empowerment model (FCEM) is an Iranian model that is based on Bandura's learning theory to promote the health status of patients with chronic diseases (15).

## Objective

Hence, CR serves as a vital tool in addressing the global burden of CVD (16). However, despite recommendations, the rates of participation and adherence in this program are low especially in low- and middle-income countries (LMICs). The reasons why CR is still unused include geographic access, cost, organizational and patient factors, and poor education and understanding of the nature of CR and the associated benefits (17). Furthermore, there is a limited understanding regarding the impact of the FCEM on the improvement of psychological symptoms in patients with post-MI. Thus, this study aimed to assess the effectiveness of the FCEM on the psychological improvement of patients with MI.

## Methods

### Study Design

This research was a randomized controlled-blind trial in patients with MI in the Cardiac Care Unit (CCU) of an educational hospital from June 2012 to January 2015. The study was approved by the institutional investigative review board at Baqiyatallah University of Medical Sciences and Tarbiat Modares University and registered with the ethical code (IR.BMSU.REC.1399.482). The incorporation criteria of this investigation were as follows: the age ranged from 45 to 85 years, ability and willingness to give knowledgeable consent, the readiness of the designee to cooperate, the ability to read, write, and fill out the questionnaire, confirmed AMI, and first hospitalization for AMI. The MI was recognized following fixed criteria, such as serum tests (e.g., troponin and creatinine kinase (CK-MB), clinical symptoms, and individual differences on the ECG). The patients had not received any CR plans beforehand. Although the patients were informed of rehabilitation of cardiac, they did not receive the details. Subjects and their designee were registered as a study unit. Power analysis estimated thirty-two patient-designee units in each group to obtain a 95% confidence level (CI) and 90% power. Attendance of the patients was recorded through convenient sampling. A statistics expert who had no clinical engagement in the experiment of the present study performed block randomization using computer-generated random numbers. The clinical supervisor of the hospital, who was not included in the recruiting process, conducted the allocation consignment. All patients, family members and friends, nurses, and data analysts were blind to the allocation process.

### Intervention

The intervention in the present study had three phases: pre-intervention, intervention, and post-intervention. Throughout the pre-intervention stage, patients filled out the questionnaires concerning their QoL, perceived stress, and anxiety. The rehabilitation program was formulated by taking into account the patient's recognized strengths and weaknesses. Patients call their study nurse every 2 days after being discharged to report any problems. Subjects were assessed weekly by their original cardiologist for 30 days. As designated, the patient's investigations involved history and physical tests, ECG, echocardiogram, and laboratory examinations. If patients had encountered any difficulty or complications, they could have informed researchers and referred to their cardiologist or care provider for evaluation. The FCEM was employed in the intervention group in four stages. Stage 1 was cognition and awareness. The patient's perception of sickness severity and perceived sensitivity, or the degree to which they felt threatened by their condition, were assessed. This assessment was achieved using 3–5 group sessions of 45–60 min; each group consisted of 3–5 people. The session's content detailed evaluations of the participant's physical and psychological status and also their view of the nature, description, risk factors, signs, medical and nursing care, and difficulties that are occurring after the MI. In stage 2, patients' expectations were examined over a 1-h session (18). Groups of 3–4 patients shared and learned from each other under the supervision and management of the principal researcher.

In stage 3, the level of patient acceptance was evaluated by utilizing an educational assistance program through group discussion. Patients used adequate problem-solving findings in the early step to reach a practical solution. Stage 4 included formative and summative evaluations (18). The formative evaluation aimed to inspire patients to internalize their control locus by understanding their self-empowerment (emphasizing self-responsibility regarding their wellbeing). Summative assessments were conducted to estimate the intervention effect on health-related QoL (HRQoL) dimensions, perceived stress, and anxiety. Phase 3 started 90 days following pre-intervention. QoL dimensions, perceived stress, and anxiety were evaluated throughout eight follow-up sessions at 3-month intervals to assess the durability and stability of patient empowerment. In this phase, patients were engaged in 21 support group webinars. Topics for the webinars included getting back to work, intimate relationships, nutrition, sleep health, smoking, exercise leisure activities, and testing or laboratory issues. Patients had options for attending the follow-up meetings, which could be arranged through appointments at home, if possible, or *via* telephone, Viber, WhatsApp, or Skype.

### Designee Role

After obtaining the patient's consent, the designee, who can be a family member or friend, stayed with the patient during the research as a study unit. The designee accompanied the patient in the training sessions related to stages 3 and 4; it is noteworthy that the patient's accompany in the second stage was optional. The designee and the patient received similar information in shared sessions. Upon the patient's request, four designees were allowed to attend the training sessions. In stage 3, the designee received educational materials and contents related to the patient. In stage 4, the designee performed the evaluations for the patient. Moreover, the designee provided a further report about the patient's status at home and current condition. Each patient was assigned a code. Anonymous information was transferred to researchers by telephone, mail, encrypted email, or manually from the designee.

### The Plan of Rehabilitation

Every patient had the same inpatient recovery plans. Patients in the FCEM group exercised daily for 0–2 h a day between 8 and 10 a.m. Exercises included walking, cycling, swimming, jogging, or other activities based on patient choice or resource availability. Designee controlled patients' daily exercise. Researchers randomly accompanied sessions in an unknown manner. It was possible to perform a physical therapy meeting at the researcher's request. This process was not routine, though. Exercise information was obtained separately from the patient and their designee every week (statistics κ = 0.9). Usual care involves knowledge of smoking suspension and schooling on meal choice. Patients had some printed materials, and the dietician evaluations were also available upon request. The patients in the control group had been provided with the same education and printed materials during the inpatient program. Patients exercised daily, at any time, for ≤ 2 h according to patient tolerance. Family members supervised the sessions. Researchers did not accompany sessions. Exercise information was separately obtained weekly from the patient and their designee (κ statistic = 0.4). Regular care included guidance on smoking suspension and meal choice. Patients were given printed materials, with dietician evaluations obtainable upon request.

### Data Collection

Four questionnaires were used to collect the data, such as the demographic questionnaire, the Short-Form Health Survey (SF-36), the 14-item Perceived Stress Questionnaire (PSQ-14), and the State-Trait Anxiety questionnaire. The SF-36 questionnaire has eight fields: physical functioning, physical role limitation, social functioning, bodily pain, mental health, mental role limitation, vitality, and general health. Each field's scoring was assessed separately, and scores varied from 0 (the worst) to 100 (the best)(19). An SF-36 questionnaire is a confirmed tool whose reliability in the present research was estimated by test-retest and Cronbach's α at 0.90 and 0.93, respectively. The PSQ-14 scores were achieved by reversing the seven positive items, such as 0 = 4, 1 = 3, 2 = 2, 3 = 1, and 4 = 0, then summing over all 14 items. Final scores vary from 14 to 70 (20, 21). The PSQ-14 reliability was evaluated in this research by test-retest and Cronbach's α at 0.93 and 0.92, respectively. Patient anxiety was assessed through the State-Trait Anxiety questionnaire. This tool has 20 items for assessing trait anxiety and state anxiety. All items are rated on a four-point scale ranging from very low (1 point) to very high (4 points). Higher scores indicate a greater level of anxiety (22, 23). The reliability of the State-Trait Anxiety questionnaire was assessed in this study by test-retest and Cronbach's α at 0.89 and 0.90, respectively.

### Data Analysis

The following multivariate mixed-effects model was used to simultaneously investigate the effect of the intervention group on study outcomes across the time:


$$\begin{array}{lll} \;y_{i}(t) & = & \eta_{i}(t)+\varepsilon_{i}(t)\\ =\beta_{0}+\beta_{1}Group & + & \beta_{2}Time+\beta_{3i}Z_{i}+b_{0i}+b_{1i}Time+\varepsilon_{i}(t)\\ \;b_{ri}\; & \sim & \;N(0,\;\sigma_{r}{}^{2})\\ \;and\;\varepsilon_{i}(t)\; & \sim & \;N(0,\;\sigma_{\varepsilon }{}^{2}) \end{array}$$

The β_3*i*_ is the fixed coefficient of adjusted confounders (*Z*_*i*_) in the model, such as body mass index (BMI), gender, age, job, education, number of families, and location, and the b_ri_ is random coefficient. Model parameters were estimated using the Markov chain Monte Carlo (MCMC) algorithm in 28,000 iterations, 3,000 burn-ins, and thinning of 50. All analyses were performed using the JMbayes package in R programming software v4.1.0. A *p*-value <0.05 (typically ≤ 0.05) was considered statistically significant.

## Results

Among all the participants in this study, 24 (34.3%) were women, with a total mean ± standard deviation (SD) of 61.40 ± 12.83 and 24.87 ± 3.80 for age and BMI, respectively (Table 1). There were no missing data, and complete outcome measurements of all eight sessions were involved in the analysis. According to the first model's results, the effect of the intervention group was significant during the follow-up for all of the outcomes. The individuals who were in the FCEM group had a significant higher mean level of perceived stress (β = 28.80, 95% CI = [24.40, 32.70]), state anxiety (β = 16.20, 95% CI = [7.88, 23.93]), trait anxiety (β = 3.6,499, 95% CI = [1.3,218, 5.7,794]), physical (β = 38.54, 95% CI = [32.02, 47.70]), and mental QoL (β = 42.14, 95% CI = [35.54, 49.47]). However, a significant increase in time variable was determined only for perceived stress (β = 1.11, 95% CI = [0.80, 1.41]) and state anxiety (β = 0.45, 95% CI = [0.22, 0.71]; Table 2).

**Table 1:** Demographics and baseline characteristics.

**Variable**	**Total (*n* = 70)**	**FCEM (n=35)**	**Control (*n* = 35)**	***P*** **-value**
Age	61.40 ± 12.83	62.00 ± 14.18	60.80 ± 11.51	0.699
BMI	24.87 ± 3.80	24.70 ± 3.5	25.3 ± 4.12	0.717
Family number	5.37 ± 1.91	5.2 ± 1.94	5.54 ± 1.91	0.454
Perceived stress	34.03 ± 3.34	34.57 ± 3.83	33.49 ± 2.70	0.176
State anxiety	55.21 ± 6.69	55.37 ± 7.21	55.06 ± 6.23	0.846
Trait anxiety	53.96 ± 4.11	54.49 ± 4.35	53.43 ± 3.84	0.285
Physical QoL	51.64 ± 7.89	52.66 ± 7.77	50.63 ± 7.98	0.285
Mental QoL	52.66 ± 8.73	51.91 ± 8.44	53.40 ± 9.08	0.481
Gender				
Male	46 (65.7%)	22 (62.9%)	24 (68.6%)	0.116
Female	24 (34.3%)	13 (37.1%)	11 (31.4%)	
Marital status				
Single	1 (1.4%)	1 (2.9%)	0 (0.00%)	NA
Married	69 (98.6%)	34 (97.1%)	35 (100.0%)	
Location				
City	36 (51.4%)	20 (57.1%)	16 (45.7%)	0.557
Countryside	34 (48.6%)	15 (42.9%)	19 (54.3%)	
Job				
Clerk	12 (17.1%)	4 (11.4%)	8 (22.9%)	0.260
Laborer	8 (11.4%)	4 (11.4%)	4 (11.4%)	
Housekeeper	23 (32.9%)	13 (37.1%)	10 (28.6%)	
Unemployed	3 (4.3%)	1 (2.9%)	2 (5.7%)	
Retired	11 (15.7%)	5 (14.3%)	6 (17.1%)	
Non-governmental	13 (18.6%)	8 (22.9%)	5 (14.3%)	
Education				
Primary	19 (27.1%)	8 (22.9%)	11 (31.4%)	0.066
Secondary	30 (42.9%)	17 (48.6%)	13 (37.1%)	
High/undergraduate	21 (30.0%)	10 (28.6%)	11 (31.4%)	

*Values are mean ± standard deviation (SD) or number (percent)*.

**Table 2:** Effectiveness of family-centered empowerment model (FCEM) using the Bayesian multivariate mixed-effects model.

**Outcome**	**Predictor**	**Posterior mean**	**Standard deviation**	**Standard error**	**Credible interval**		**P-value**
					**2.5%**	**97.5%**	
Perceived stress	Time	1.1,098	0.1,525	0.0,089	0.8030	1.4,142	***<0.001***
	Group	28.8,020	2.1,301	0.2,286	24.3985	32.6,965	***<0.001***
State anxiety	Time	0.4,481	0.1,203	0.0,050	0.2,176	0.7,150	***<0.001***
	Group	16.2,076	4.1,618	0.4,082	7.8,821	23.9,306	***<0.001***
Trait anxiety	Time	−0.0,515	0.0,527	0.0,055	−0.1,505	0.0,580	0.306
	Group	3.6,499	1.1,751	0.0,536	1.3218	5.7,794	**0.006**
Physical QoL	Time	0.2,965	0.2,691	0.0,151	−0.2,242	0.8,041	0.276
	Group	38.5,362	4.2,624	0.7,714	32.0,172	47.7,082	***<0.001***
Mental QoL	Time	0.1,556	0.2,825	0.0,133	−0.3,948	0.6,954	0.572
	Group	42.1,384	3.5,040	0.5237	35.5,423	49.4,673	***<0.001***

*Adjusted for age, gender, BMI, job, education, family number, and location. The bold and italic values are considered as statistically significant*.

**Figure 1:**
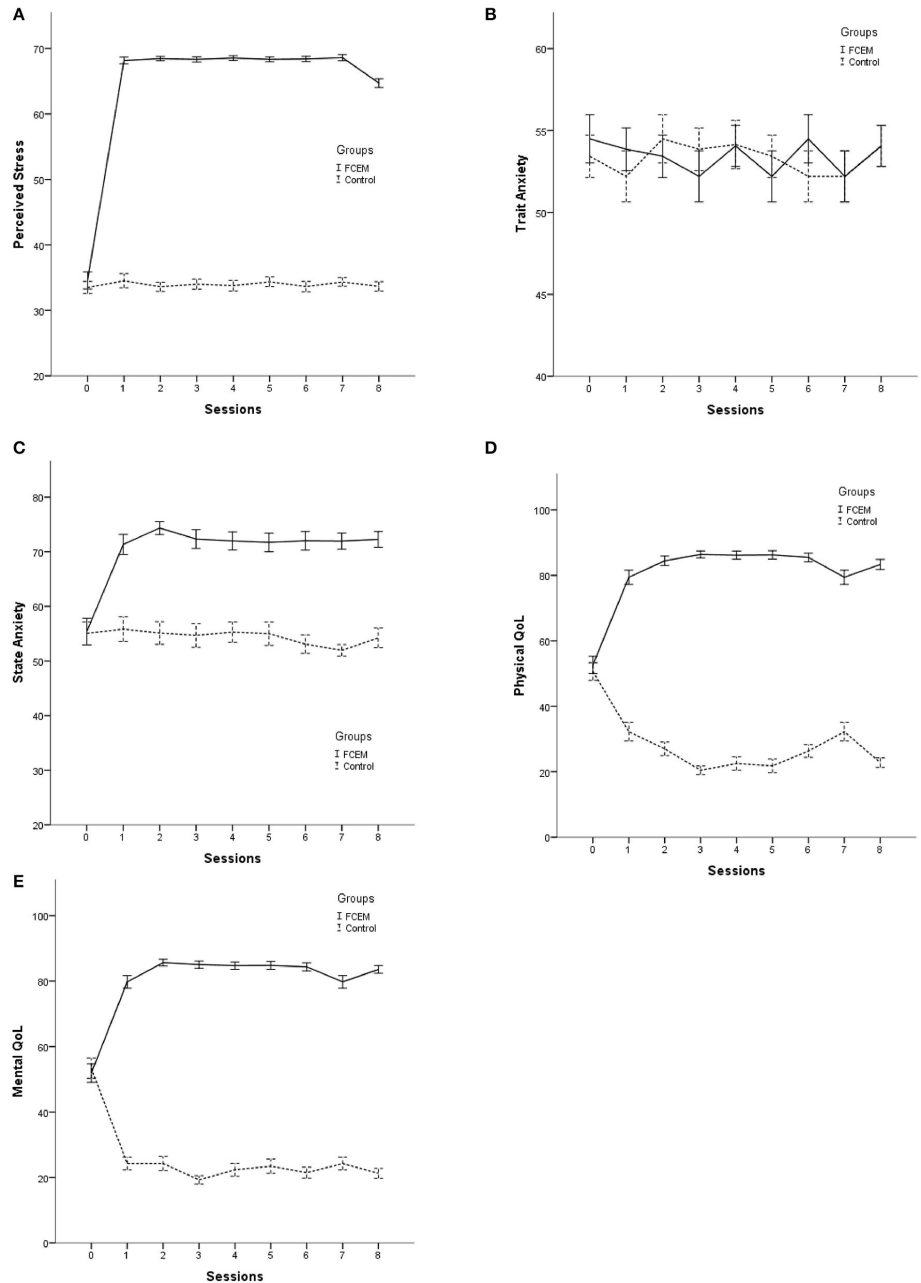
The trend of study outcomes across the follow-up sessions according to the study groups. **(A)** Perceived stress assessment; **(B)** Trait anxiety assessment; **(C)** State anxiety assessment; **(D)** Physical dimension of quality of life; and **(E)** Mental dimension of quality of life.

Meanwhile, further analysis through the second model revealed that the intervention had not significantly improved all aspects of QoL. That is, the individuals in the FCEM group had a significant higher mean level of general health (β = 31.64, 95% CI = [16.39, 44.53]) in the physical dimension, and vitality (β = 15.04, 95% CI = [0.82, 28.36]), mental role limitation (β = 21.84, 95% CI = [3.95, 40.06]), and mental health (β = 18.16, 95% CI = [6.01, 29.71]) in the mental dimension. The effect of the intervention on the other subscales was not significant. Moreover, the effect of time was only significant for vitality and social functioning (Table 3). The trend of study outcomes across the follow-up sessions according to the study groups are illustrated in Figure 1.

**Table 3:** Effectiveness of family–centered empowerment model (FCEM) using the Bayesian multivariate mixed–effects model in different quality of life subscales.

**Outcome**	**Predictor**	**Posterior mean**	**Standard deviation**	**Standard error**	**Credible interval**		**P–value**
					**2.5%**	**97.5%**	
**Physical dimension**							
Physical functioning	Time	0.2,785	0.2,687	0.0,093	−0.2549	0.7,740	0.324
	Group	11.2,419	7.0,012	0.4,635	−2.7076	24.6,365	0.122
Physical role limitation	Time	0.2,033	0.4,469	0.0,193	−0.7146	1.0,284	0.630
	Group	11.2,770	8.5,648	0.3,936	−5.7407	28.2,580	0.194
Bodily pain	Time	−0.5,614	0.3,123	0.0,118	−1.1710	0.0,471	0.064
	Group	7.1,780	8.3,271	0.4,831	−9.2237	23.1,481	0.398
General health	Time	−0.3,858	0.3,097	0.0,118	−1.0022	0.1,937	0.196
	Group	31.6,414	7.0,733	0.6,124	16.3891	44.5,323	***<0.001***
**Mental dimension**							
Vitality	Time	−0.6,070	0.2,817	0.0,089	−1.1,509	−0.0,816	***0.030***
	Group	15.0,367	7.0,142	0.3,806	0.8,172	28.3,614	***0.040***
Social functioning	Time	−0.6,958	0.3,293	0.0,104	−1.3,430	−0.0,811	***0.018***
	Group	13.4,486	8.9,295	0.3,742	−4.4,469	30.8,094	0.136
Mental role limitation	Time	−0.2,285	0.5,283	0.0,264	−1.2,901	0.7,616	0.684
	Group	21.8,421	9.0,435	0.8,136	3.9,470	40.0,627	***0.010***
Mental health	Time	−0.2,402	0.2,570	0.0,093	−0.7,275	0.2,684	0.360
	Group	18.1,587	6.1,368	0.3,990	6.0,082	29.7,101	*** <0.001***

*Adjusted for age, gender, BMI, job, education, family number, and location. The bold and italic values are considered as statistically significant*.

## Discussion

The present study aimed to investigate the effectiveness of the FCEM on the psychological improvement of patients with MI. The effect of the intervention group was significant throughout the follow-up for all of the outcomes, according to the results of the first model. Individuals in the FCEM group had a considerably higher mean level of perceived stress, state anxiety, trait anxiety, and physical and mental QoL. However, a significant increase in time variables was found only for perceived stress and state anxiety. These results were compatible with the findings of other studies. The results of the Etemadifard et al. showed that a family-centered intervention plan can decrease anxiety and stress in caregivers due to the simplicity, feasibility, and usefulness of the intervention (24). In addition, Chauvet-Gelinier et al. showed that CR seems to be a crucial step in improving patients' outcomes by helping them to understand the influence of psychobiological risk factors and building strategies to manage daily stress (4). The previous studies showed that the symptoms of anxiety and stress in patients with MI are intimately associated with survival in the first year after admission (15, 16). Manoj et al. showed that people with an increased level of depression, anxiety, and stress are at increased risk of MI when compared with people without any significant risk factors of MI (25). The symptoms of anxiety and stress were slightly connected to the AMI risk and lower life quality in 10 years (4, 14). Anxiety changes the cardiac rhythm and increases the risk of coronary artery spasms, leading to atherosclerosis and coronary artery disease eventually (26). Severe stress increases atherosclerosis and the risk of coronary artery occlusion. The body responds to stress by releasing an excessive amount of norepinephrine and neurotransmitters, which damages myocardial nerve endings and increases the susceptibility to MI (4). Therefore, following the first time AMI symptoms, patients require immediate evaluation and therapy, and early CR may play an essential role in post-MI anxiety or stress. As a result, applying the FCEM will lead to the psychological improvement of the patient. The participation of family members as the most crucial external factor in reducing psychological problems is essential.

Further analysis through the second model revealed that not all aspects of QoL had improved by the intervention. The individuals in the FCEM group had a higher level of general health in the vitality, physical dimension, mental role limitation, and mental health. Faramarzi et al. confirmed that the FCEM in angina patients had developed their life quality (27). Patient- and family-centered care is among the high-quality programs in healthcare settings. It has already been confirmed that the FCEM model may be available for cardiac applications.

Furthermore, CR has been described as applicable to patients undergoing coronary artery bypass surgery (28). The study has shown that the CR can lead to an increase in anxiety and stress among patients with MI and improve their HRQoL (29). Anxiety symptoms were not significantly associated with education, gender, MI, and prior AMI history, except for risk factors related to CHDs, such as hypertension, hyperlipidemia, smoking, and physical activity to combat anxiety (30). Therefore, anxiety is more likely linked to currently signed reminders of full health (31). Anxiety and stress affect MI disorders by mechanisms central to the hypothalamic-pituitary-adrenal (HPA) and autonomic nervous systems and can increase secretion of catecholamine sympathetic nerve activity, activating platelets and inflammation eventually (32).

Anxiety and stress are commonly undervalued among patients with MI. Consequently, the primary concern is to promote awareness among health workers regarding this concurrent. Likely, one research has explained that patients with anxiety are more prone to CR, leading to a better diagnosis and optimal rehabilitation (33). Besides the FCEM, entirely psychological treatment, such as problem-solving therapy (PST), cognitive-behavioral therapy (CBT), possibly psychodynamic psychotherapy, interpersonal psychotherapy (IPT), and pharmacotherapies, is practical for treating anxiety and stress in patients. These interventions are all part of CR (34).

We found improvements in physical health, mental health, and QoL in those patients receiving home CR using the FCEM as compared to the group control. The reasons for such improvement are most likely multifactorial and may include patient encouragement, greater patient understanding, positive reinforcement, and a sense of accountability, which is consistent with other studies (11, 35, 36).

### Limitations of the Study

The limitations of this study may include uncontrollable intervention variables, such as different psychological characteristics, cultural and social contexts, patients' interpersonal interactions, and differences in the study units' motivations and personal interests. Moreover, since the accuracy of remotely performed evaluations is generally inferior to in-person examinations, post-intervention assessments could be performed remotely and through different media to achieve the maximum effectiveness and accuracy. However, the use of different media increases the heterogeneity of collected data. Therefore, it is recommended that in future research in this context, post-intervention assessments to be performed through in-person examinations if possible or at least through one unique media.

## Conclusion

In conclusion, the FCEM can be a helpful treatment mechanism for anxiety and stress symptoms in patients with post-MI. Moreover, initial CR in these patients with anxiety or stress is necessary to overcome life quality, morbidity, and mortality disadvantageous. Prospective investigations are required to study the influence of CR on anxiety and stress at various times throughout mostly recovery prediction, which may lead to further clinical rehabilitation.

## Data Availability Statement

The original contributions presented in the study are included in the article/Supplementary Material, further inquiries can be directed to the corresponding author/s.

## Ethics Statement

The studies involving human participants were reviewed and approved by Institutional investigative review board at Baqiyatallah University of Medical Sciences. The patients/participants provided their written informed consent to participate in this study. Written informed consent was obtained from the individual(s) for the publication of any potentially identifiable images or data included in this article.

## Author Contributions

AV-A and MR concepted and designed the project. AV-A, MR, MG, KH, NS-K, and HR acquisited the data. MR and NS-K analyzed and interpreted the data. All authors contributed to the article and approved the submitted version.

## Conflict of Interest

The authors declare that the research was conducted in the absence of any commercial or financial relationships that could be construed as a potential conflict of interest.

## Publisher's Note

All claims expressed in this article are solely those of the authors and do not necessarily represent those of their affiliated organizations, or those of the publisher, the editors and the reviewers. Any product that may be evaluated in this article, or claim that may be made by its manufacturer, is not guaranteed or endorsed by the publisher.
